# Efficacy and safety of cyclosporine a for patients with steroid-resistant nephrotic syndrome: a meta-analysis

**DOI:** 10.1186/s12882-019-1575-8

**Published:** 2019-10-23

**Authors:** Hong-Yan Li, Xialan Zhang, Tianbiao Zhou, Zhiqing Zhong, Hongzhen Zhong

**Affiliations:** 1 Department of Nephrology, Huadu District People’s Hospital of Guangzhou, Southern Medical University, Guangzhou, China; 2 Department of Obestetrics and Gynecology, the Second Affiliated Hospital of Shantou University Medical College, Shantou, 515041 China; 3 Department of Nephrology, the Second Affiliated Hospital of Shantou University Medical College, Shantou, 515041 China

**Keywords:** Cyclosporine a (CsA), Steroid-resistant nephrotic syndrome (SRNS), Complete remission (CR), Total remission (TR), Meta-analysis

## Abstract

**Background:**

The purpose of this study was to determine efficacy and safety of cyclosporine A (CsA) for patients with steroid-resistant nephrotic syndrome (SRNS).

**Methods:**

The Cochrane Library and PubMed were searched to extract the associated studies on Oct 10, 2018, and the meta-analysis method was used to pool and analyze the applicable investigations included in this study. The P(opulation) I(ntervention) C(omparison) O(utcome) of the study were defined as follows: P: Patients with SRNS; I: treated with CsA, cyclophosphamide (CYC), tacrolimus (TAC) or placebo/not treatment (P/NT); C: CsA vs. placebo/nontreatment (P/NT), CsA vs. CYC, CsA vs. TAC; O: complete remission (CR), total remission (TR; complete or partial remission (PR)), urine erythrocyte number, proteinuria levels, albumin, proteinuria, serum creatinine, and plasma cholesterol, etc. Data were extracted and pooled using RevMan 5.3.

**Results:**

In the therapeutic regimen of CsA vs. placebo/nontreatment (P/NT), the results indicated that the CsA group had high values of CR, TR, and low values of proteinuria, serum creatinine, and plasma cholesterol when compared with those in the placebo group. In comparing CsA vs. cyclophosphamide (CYC), the results indicated that the CsA group had higher TR than the CYC group. In comparing CsA vs. tacrolimus (TAC), the results revealed insignificant differences in CR, and TR between the CsA and TAC groups. The safety of CsA was also assessed. The incidence of gum hyperplasia in CsA group was higher than that in the P/NT group, with no differences in incidence of infections or hypertension between CsA and P/NT groups. There was no difference in the incidence of hypertension between the CsA and TAC groups.

**Conclusions:**

CsA is an effective and safe agent in the therapy of patients with SRNS.

## Background

Nephrotic syndrome (NS), characterized by hypoalbuminemia, massive proteinuria, peripheral edema, and hyperlipidemia, is a major cause of end-stage renal disease (ESRD), and related damage of the glomerular filtration barrier [1–3]. Based on the response to steroid therapy, NS is classified as steroid-sensitive nephrotic syndrome (SSNS, approximately 50% of SSNS patients develop frequently-relapsing nephrotic syndrome or steroid-dependent nephrotic syndrome), or steroid-resistant nephrotic syndrome (SRNS) [1, 4–6]. Patients who do not enter remission after administration of daily prednisolone for 4 weeks are regarded as SRNS [7]. SRNS is regarded as one of the most common causes of the development of ESRD in children [8]. The current therapeutic options for SRNS are often ineffective, it frequently progresses to a loss of kidney function, and treatment is often complicated by significant toxicity associated morbidities, mortality, and cost [1, 8].

Cyclosporine A (CsA) is one of the most widely used immunosuppressants in organ transplantation and in the treatment of various immunological diseases [9, 10]. CsA is frequently used to treat SRNS and can induce remission [11, 12]. However, CsA also exerts nephrotoxic effects, as demonstrated by increased tubulointerstitial fibrosis, inflammation and podocyte damage [13, 14]. In the current study, we performed a meta-analysis to assess the safety and efficacy of CsA in the treatment of patients with SRNS.

## Methods

### Data sources and search strategy

The systematic search strategy was conducted in the Cochrane Library and PubMed without language restrictions, from inception to Oct 10, 2018. We conducted searches by using the search strategy: cyclosporine AND (nephrotic syndrome OR glomerulonephritis membranoproliferative OR focal segment glomerulosclerosis OR minimal change nephrotic syndrome OR membranoproliferative glomerulonephritis). We also checked the references cited in the published studies for additional studies.

### Inclusion and exclusion criteria

#### Inclusion criteria

In this study, the inclusion criteria were as follows: (1) investigation type: randomized controlled trials; (2) object of the study: patients were diagnosed with NS and the NS was resistant to the steroid treatment; (3) type of interventions: treatment regimens based on CsA, the controls should have been treated with another immunotherapy or placebo.

#### Exclusion criteria

Exclusion criteria for the study were as follows: (1) Reviews, case reports, letters, systematic reviews, and meta-analysis; (2) Patients with nephrotic syndrome were sensitive to steroid or dependent to steroid; (3) studies that do not contain different therapeutic regimens; (4) the diagnostic criteria were not clear.

#### Analyzed outcomes

Efficacy of CsA: primary outcomes were complete remission (CR) and total remission (TR; CR or partial remission (PR)). The secondary outcomes were biological indicators including proteinuria levels, serum creatinine, serum albumin and plasma cholesterol.

Safety of CsA: adverse events including infection, hypertension and gum hyperplasia.

The CR was defined as proteinuria < 4 mg/m^2^/hr. (children) or 0.2 g/day (adults), for three non-consecutive days. PR was defined as the proteinuria < 40 mg/m^2^/hr. (children) or 3.5 g/day (adults) for three different non-consecutive days.

#### Data collection

According to the predetermined inclusion criteria, two independent reviewers scanned the titles and abstracts of the included records. Full texts of potentially literature were read for further screening. Discordant opinions were discussed and resolved by other reviewers.

The extraction data included the (1) the first author and publication year, (2) study design features, (3) baseline characteristics of study participants, and (4) study outcomes (e.g., efficacy and safety outcomes). The P(opulation) I(ntervention) C(omparison) O(utcome) of the study were defined as follows: P: Patients with SRNS; I: treated with CsA, CYC, tacrolimus (TAC) or placebo/not treatment (P/NT); C: CsA vs. placebo/nontreatment (P/NT), CsA vs. cyclophosphamide (CYC), CsA vs. TAC; O: CR, TR, urine erythrocyte number, proteinuria levels, albumin, proteinuria, serum creatinine, and plasma cholesterol, etc.

#### Quality assessment

Two abstractors independently evaluated the methodological quality of all the eligible clinical trials according to the Modified Jadad Scale[15]. The studies were scored by answering the following questions:

Randomization:

1. Was the trial random?
2. Was the randomization procedure adequately explained?

Allocation concealment:

1. Did the trial use a random assignment method?
2. Was the allocation concealment appropriate so that the clinicians and the subjects could not predict how the sequence would be assigned?

Blinding method:

1. Was the trial double-blind?
2. Did the trial use a placebo or similar methods?

Withdrawals and dropouts:

1. Were the numbers and reasons for withdrawals and dropouts adequately explained?

If the answer to each question was YES, the study would get 1 point; if NO, the study would get 0 point. A score of more than 3 was considered as high quality.

### Statistical analysis

The data were extracted from the included literature, and the results were evaluated using Review Manager Version 5.3 software (Revman the Cochrane Collaboration; England). Continuous data were expressed using weighted mean differences (WMDs), and dichotomous data were expressed using the odds ratio (OR). 95% confidence intervals (95% CI) with the Mantel-Haenszel (M-H) method were used for the included studies. Heterogeneity was analyzed using I^2^ statistics and calculated for all the meta-analyses. On the basis test of the heterogeneity, when the *p*-value less than 0.1 or the I^2^ < 50%, a fixed effect model was used. Otherwise, the results were counted using a random effects model, and a *p*-value < 0.05 denoted significance.

## Results

### Search results

Seven randomized controlled trials [16–22] related to CsA for SRNS were included (Table 1), three studies [17, 19, 21] for CsA vs. Placebo and two studies [16, 22] for CsA vs. TAC (Table 1). The quality assessment details, obtained using the Modified Jadad Scale, are presented in Table 2.

**Table 1:** Characteristics of the studies included in this meta-analysis

Author, year	Study design	Treatment strategies	Detailed scheme	Patient characteristics	Main Outcome Measures	adverse events
Garin 1988	Single center, cross-over, randomized clinical trial	CsA vs. P/NT	In CsA group, the initial dosage was 5 mg/kg/d, and then sustained 200 ng/ml or less. The total treatment course was 8 weeks. In control group, any immunosuppressive agent was not allowed.	Six male and two females with idiopathic, SRNS were enrolled. The median age was 12 (3, 18) years. Four patients had MCD and the other four were diagnosed with FSGS. All the patients were Children. Initially, four patients in group of CsA and four patients in P/NT group.	Urinary protein excretion values, creatinine clearance values, serum albumin values, etc.	Hypertension, renal function deterioration, liver function disorder, etc.
Ponticelli 1993	Multicenter randomized clinical trial	CsA vs. P/NT	CsA was administered orally and the initial dose was divided into two doses (6 mg/kg/day for children and 5 mg/kg/day for adults). The CsA level was maintained between 250 and 600 ng/ml, and CsA discontinued after six months. For patients who responded, the CsA dose was reduced by 25% every two months, so that CsA treatment was stopped by the end of the year. Patients in control group were given only supportive treatment for one year.	The following characteristics met included criterion: 1) Patients had nephrotic syndrome; 2) The creatinine clearance was more than 80 ml/min/1.73m^2^ for children 60 ml/min/1.73m^2^ for adults; 3) The renal biopsies showed either MCD or FSGS. The patients included children and adults. 22 patients were in CsA group and 19 patients were in P/NT group.	CR, TR, proteinuria, serum creatinine, creatinine clearance values, serum urea, serum proteins, serum albumin values, plasma cholesterol, etc.	Infections, gum hyperplasia, hypertrichosis, transient gastric discomfort, conjugated bilirubinemia, headache, bronchospasm, paresthesia, etc.
Lieberman 1996	Multicenter randomized clinical trial	CsA vs. P/NT	CsA was administered as the 100 mg/ml suspension. The initial dose was 3 mg/kg of CsA twice daily as to attaining a target CsA level within the range of 300 to 500 ng/mL. Placebo patients were received a vehicle control.	Age of patients was between 6 months and 21 years. 15 patients were in CsA group and 15 patients were in P/NT group.	CR, TR, proteinuria, serum creatinine, serum albumin values, plasma cholesterol, potassium, uric acid, magnesium, etc.	Infections, hypertension, gum hyperplasia, etc.
Plank 2008	Multicenter randomized clinical trial	CsA vs. CYC	The initial dose of CsA was 150 mg/m^2^ BSA twice per day in CsA group. If the proteinura decrease < 40 mg/m^2^/hour within the first 12 weeks during the CsA therapy, patients were recruited into the non-responder protocol with CsA dose increasing to 350 ng/ml (range 300–400 ng/ml). In control group, patients were administered 500 mg/m^2^ BSA CYC pulse therapy in a 4-h infusion. The infusion treatment was repeated in 4,8,12,16,24 and 36 weeks.	Patients from children with gross proteinuria > 40 mg/m^2^ BSA per hour (equivalent to 1 g/m^2^/24h) and hypoalbuminemia (< 25 g/l) were included. All patients were diagnosed as MCD, FSGS or diffuse mesangial proliferation by renal biopsy. 15 patients were in CsA group and 17 patients were in CYC group.	CR, TR, etc.	Infections, hypertension, headache, gum hyperplasia, hypertrichosis, transient gastric discomfort, etc.
Choudhry 2009	Single center Randomized clinical trial	CsA vs. TAC	The initial dose of TAC was 0.1 to 0.2 mg/kg/d or CsA at 5 to 6 mg/kg/d in two divided doses for 12-month. Patients in two groups got oral prednisolone 1 mg/kg (on alternate days) for 6 months, followed by 0.5 mg/kg for the next 6-month, and enalapril was administered at the dose of 0.2 to 0.3 mg/kg/d.	Patients with the following traits were eligible for study 1) Age ranged from 1 to 18 years with idiopathic SRNS; 2) Renal histological characteristics suggested of MCD, mesangioproliferative glomerulonephritis or FSGS. 20 patients were in CsA group and 21 patients were in TAC group.	CR, TR, etc.	Infections, hypertension, headache, gum hyperplasia, hypertrichosis, paresthesia, etc.
Geng 2018	Single center Randomized clinical trial	CsA vs. MMF	In CsA group, CsA was administered orally 3~5 mg/kg twice a day, and maintained between 80 and 120 μg/L. The maintenance dose of effective patients was 1–3 mg/(kg·d) and their induction period was reduced after 6 months. In MMF group, the dose was administered orally in 2 patterns, either 15–30 mg/(kg·d) or 800~1200 mg/(m^2^·d). The MMF was discontinued if the decreasing in proteinuria was less than 50%, or continued to treat after 6 months of induction with maintenance dose of 10–20 mg/(kg·d). The total course was 12 months.	30 pediatric patients aged from 2.1 to 17.0 years presented with SRNS. The renal histological characteristics indicated MCD, mesangioproliferative glomerulonephritis, MN or FSGS. 18 patients were in CsA group and 14 patients were in MMF group.	CR, TR, etc.	Respiratory infections, nausea, vomiting, abdominal pain, diarrhea and other gastrointestinal symptoms, etc.

Note: *SRNS* steroid-resistant nephrotic syndrome; *CsA* cyclosporine A; *TAC* tacrolimus; *CYC* cyclophosphamide; *MMF* mycophenolate mofetil; *CR* complete remission; *TR* total remission (complete or partial remission (PR)); *P/NT* placebo/nontreatment; *FSGS* focal segmental glomerulosclerosis; *MCD* minimal change nephropathy; *MN* membranous nephropathy; *h* hour; *NA* not available. *BSA* body surface area

**Table 2:** Quality Assessment of Included Studies (7-point)

Author, year	Type	Randomization	Concealment of allocation	Double blinding	Withdrawals and dropouts	Jadad score
Ponticelli 1993 (CsA vs. P/NT)	An open, prospective, randomized, multicentric, controlled study	According to a table of random numbers	By using sealed, completely opaque envelopes	Open-label	Yes	5
Lieberman 1996(CsA vs. P/NT)	A double-blinded, prospectively randomized, placebo-controlled trial	By following computer based random numbers	By using sequentially labelled sealed envelopes	Both the patients and their pediatric nephroboglsts were blinded as to the administered study treatment	Yes	7
Plank 2008 (CsA vs. CYC)	A controlled multicentre randomized open label trial	According to centre-specific computer-generated random lists	Describing as using allocation concealment but without any details	Open-label	Yes	5
Choudhry 2009 (CsA vs. TAC)	A nonblind, randomized controlled trial	By following computer based random numbers	By using serially numbered opaque envelopes	Open-label	No	5
Garin 2015 (CsA vs. P/NT)	A cross-over, randomized, controlled trial	Describing as a randomized trial without details	NA	NA	NA	1
Geng 2018 (CsA vs. MMF)	A prospective, randomized controlled clinical trial	According to a table of random numbers	NA	NA	Yes	3

Note: *CsA* cyclosporine A; *CTX* cyclophosphamide; *TAC* tacrolimus; *MMF* mycophenolate mofetil; *NA* not available

### The comparison of CsA vs. placebo/nontreatment (P/NT)

**Fig. 1:**
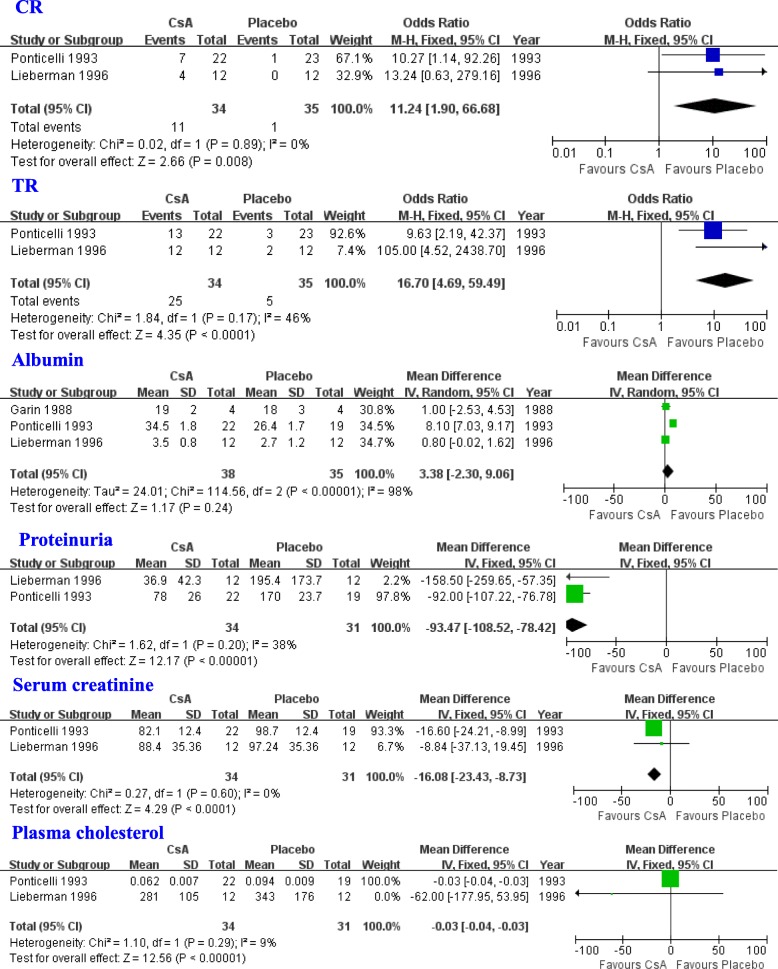
Assessment of the efficacy of CsA in patients with SRNS. SRNS: steroid-resistant nephrotic syndrome; CsA: cyclosporine A; CR: complete remission; TR: total remission, complete or partial remission, M-H: Mantel-Haenszel

Three studies [17, 19, 21] were included in the meta-analysis to assess the efficacy of CsA in patients with SRNS. The results indicated that the CsA group had a higher CR (OR = 11.24, 95% CI: 1.90–66.68, *P* = 0.008; Fig. 1), and TR (OR = 16.70, 95% CI: 4.69–59.49, *P* < 0.0001; Fig. 1). The CsA treatment group displayed elevated levels of albumin when compared with the P/NT group, although this was not statistically different (WMD = 3.38, 95% CI: − 2.30-9.06, *P* = 0.24; Fig. 1). The CsA group had lower levels of proteinuria, serum creatinine, and plasma cholesterol when compared with the P/NT group (proteinuria: WMD = -93.47, 95% CI: − 108.52 to − 78.42, *P* < 0.00001; serum creatinine: WMD = -16.08, 95%CI: − 23.43 to − 8.73, *P* < 0.0001; plasma cholesterol: WMD = -0.03, 95% CI: − 0.04 to − 0.03, *P* < 0.00001; Fig. 1).

The safety of CsA was also assessed in patients with SRNS. The incidence rate of gum hyperplasia in the CsA group was higher than that in P/NT group (OR = 13.50, 95% CI: 1.66–109.84, *P* = 0.01). The incidence rates of infection or hypertension were similar between the CsA and P/NT groups (infections: 95% CI: 0.24–2.33, OR = 0.75, *P* = 0.62; hypertension: 95% CI: 0.12–8.56, OR = 1.00, *P* = 1.00).

### Comparing CsA vs. CYC

One study [20] including two comparisons was considered in the meta-analysis to assess the efficacy of CsA in patients with SRNS compared with CYC. The results indicated that the CsA group had a higher TR than the CYC group (OR = 12.83, 95% CI: 3.85–42.81, *P* < 0.0001). The CsA group had a higher CR than the CYC group, although there was no statistical difference (OR = 1.59, 95% CI: 0.33–7.76, *P* = 0.57).

### Comparing CsA vs. TAC

Two studies [16, 22] of CsA vs. TAC were included into the meta-analysis to assess the efficacy of CsA in patients with SRNS. There were no significant group differences in CR or TR (CR: OR = 1.71, 95%CI: 0.58–5.04, *P* = 0.33; TR: OR = 0.50, 95% CI: 0.10–2.44, *P* = 0.39).

*The safety of CsA was also assessed in patients with SRNS. There were no significant group differences in rates of hypertension (OR = 4.51, 95% CI: 0.21–96.06, P = 0.33).*

## Discussion

In this systemic review and meta-analysis, we assessed the efficacy of CsA in the treatment of SRNS as well as the safety of CsA. In comparing CsA vs. placebo, the results indicated that CsA treatment increases CR, and TR and decreases proteinuria, serum creatinine, and plasma cholesterol. However, the patients from two selected studies included adults, and there was only one study on children. More studies on children or adults should be conducted to broadly assess the efficacy of CsA in the treatment of SRNS. CsA treatment did not increase rates of serious adverse events, such as infections or hypertension, but it did increase rates of gum hyperplasia. These results indicate that the CsA might be a good agent for the treatment of SRNS.

We also performed comparisons of CsA vs. CYC and CsA vs. TAC. CsA treatment results in a higher TR when compared to CYC. The CsA group also had a higher CR than the CYC group, although this difference was not significant. However, the number of included studies was small, and more investigations are needed to confirm these findings. In comparing CsA vs. TAC, the results indicated no group differences in TR, CR or adverse events. This may indicate that CsA has similar efficacy and safety to TAC. CsA and TAC are two most important members of calmodulin inhibitors, and the efficacy and safety may be due to this similarity.

The number of included studies used to assess differences between CsA and mycophenolate mofetil (MMF) in the treatment of SRNS in the current analysis was small. Geng et al. [18] compared the efficacy and safety of CsA versus MMF in the treatment of children suffering from primary refractory nephrotic syndrome, and reported that CsA was superior to MMF in preventing relapses in children with frequently relapsing nephrotic syndrome and inducing complete remission in SRNS patients. Although most patients with SRNS are able to tolerate CsA and MMF, the toxicity and safety of CsA should be monitored closely. More RCTs should be conducted to assess the differences between CsA and MMF in the treatment of SRNS.

There have been two previous two meta-analyses assessing the efficacy of CsA in the treatment of SRNS. Jiang et al. [23] conducted a meta-analysis to detect the efficacy of CsA, TAC, and CYC in treating SRNS, and included four studies of CsA. They reported that CsA has superior efficacy compared to CYC and placebo. Hodson et al. [24] reported that CsA significantly increases the number of children who achieve CR when compared with P/NT. In our meta-analysis, we also assessed the safety of CsA in the treatment of SRNS, and found it to be a safe and effective immunosuppressive agent in the treatment of children with SRNS.

We used the modified Jadad Scale to score the included trials and observed that only one study [17] was scored less than 3. We excluded, performed the meta-analysis again, and the results were similar to the initial analysis. However, the number of included studies in the current analyses was small, and additional analyses should be conducted to confirm the present findings.

There were some limitations in the current meta-analysis. Most of studies were of children, but some studies included both children and adults. Independent assessment of the efficacy and safety of CsA in the treatment of SRNS in children and adults is needed. The target renal histological characteristics were MCD, mesangioproliferative glomerulonephritis, MN or FSGS, but not all the studies included these histological characteristics, which increased heterogeneity among the recruited studies.

## Conclusions

In the current meta-analysis, we conclude that CsA is an effective and safe therapy for SRNS. However, additional RCT studies are needed to thoroughly assess the role of CsA in the treatment of SRNS.

## Data Availability

All data generated or analysed during this study are included in this published article.

## Abbreviations

CI: Confidence intervals
CR: Complete remission
CsA: Cyclosporine A
M-H: Mantel-Haenszel
MMF: Mycophenolate mofetil
NS: Nephrotic syndrome
OR: Odds ratio
P/NT: Placebo/nontreatment
SRNS: Steroid-resistant nephrotic syndrome
TR: Total remission
WMDs: Weighted mean differences

## References

[1] Asharam K, Bhimma R, David VA, Coovadia HM, Qulu WP, Naicker T (2018). NPHS2 V260E is a frequent cause of steroid-resistant Nephrotic syndrome in black south African children. Kidney Int Rep.

[2] Varner JD, Chryst-Stangl M, Esezobor CI, Solarin A, Wu G, Lane B (2018). Genetic testing for steroid-resistant-Nephrotic syndrome in an outbred population. Front Pediatr.

[3] Dogra S, Kaskel F (2017). Steroid-resistant nephrotic syndrome: a persistent challenge for pediatric nephrology. Pediatr Nephrol.

[4] Querfeld U, Weber LT (2018). Mycophenolate mofetil for sustained remission in nephrotic syndrome. Pediatr Nephrol.

[5] Akchurin OM, Kaskel FJ (2013). Late steroid resistance in childhood nephrotic syndrome: do we now know more than 40 years ago?. Pediatr Nephrol.

[6] Horinouchi T, Sako M, Nakanishi K, Ishikura K, Ito S, Nakamura H (2018). Study protocol: mycophenolate mofetil as maintenance therapy after rituximab treatment for childhood-onset, complicated, frequently-relapsing nephrotic syndrome or steroid-dependent nephrotic syndrome: a multicenter double-blind, randomized, placebo-controlled trial (JSKDC07). BMC Nephrol.

[7] Tullus K, Webb H, Bagga A (2018). Management of steroid-resistant nephrotic syndrome in children and adolescents. Lancet Child Adolesc Health.

[8] Siji A, Karthik KN, Pardeshi VC, Hari PS, Vasudevan A (2018). Targeted gene panel for genetic testing of south Indian children with steroid resistant nephrotic syndrome. BMC Med Genet.

[9] Liu C, Zhu P, Fujino M, Isaka Y, Ito H, Takahashi K, et al. 5-aminolaevulinic acid (ALA), enhances heme oxygenase (HO)-1 expression and attenuates tubulointerstitial fibrosis and renal apoptosis in chronic cyclosporine nephropathy. Biochem Biophys Res Commun. 2019;508(2):583-9. 10.1016/j.bbrc.2018.11.175 30514440

[10] Groenendyk J, Paskevicius T, Urra H, Viricel C, Wang K, Barakat K (2018). Cyclosporine a binding to COX-2 reveals a novel signaling pathway that activates the IRE1alpha unfolded protein response sensor. Sci Rep.

[11] Liu Y, Yang R, Yang C, Dong S, Zhu Y, Zhao M (2018). Cyclophosphamide versus cyclosporine a therapy in steroid-resistant nephrotic syndrome: a retrospective study with a mean 5-year follow-up. J Int Med Res.

[12] Gellermann J, Ehrich JH, Querfeld U (2012). Sequential maintenance therapy with cyclosporin a and mycophenolate mofetil for sustained remission of childhood steroid-resistant nephrotic syndrome. Nephrol Dial Transplant.

[13] Loboda A, Mucha O, Podkalicka P, Sobczak M, Miksza-Cybulska A, Kaczara P, et al. Kidney injury by cyclosporine a is aggravated in heme oxygenase-1 deficient mice and involves regulation of microRNAs. Acta Biochim Pol. 2018;65(4):613-20. 10.18388/abp.2018_2658 30481230

[14] Gellermann J, Schaefer F, Querfeld U (2014). Serum suPAR levels are modulated by immunosuppressive therapy of minimal change nephrotic syndrome. Pediatr Nephrol.

[15] Li ZQ, Hu ML, Zhang C, Wang YM (2015). Efficacy and safety of tacrolimus vs. cyclophosphamide for idiopathic membranous nephropathy: a meta-analysis of Chinese adults. J Huazhong Univ Sci Technolog Med Sci.

[16] Choudhry S, Bagga A, Hari P, Sharma S, Kalaivani M, Dinda A (2009). Efficacy and safety of tacrolimus versus cyclosporine in children with steroid-resistant nephrotic syndrome: a randomized controlled trial. Am J Kidney Dis.

[17] Garin EH, Orak JK, Hiott KL, Sutherland SE (1988). Cyclosporine therapy for steroid-resistant nephrotic syndrome. A controlled study. Am J Dis Child.

[18] Geng HY, Ji LN, Chen CY, Tu J, Li HR, Bao R (2018). Mycophenolate mofetil versus cyclosporine a in children with primary refractory nephrotic syndrome. Chin J Pediatr.

[19] Lieberman KV, Tejani A (1996). A randomized double-blind placebo-controlled trial of cyclosporine in steroid-resistant idiopathic focal segmental glomerulosclerosis in children. J Am Soc Nephrol.

[20] Plank C, Kalb V, Hinkes B, Hildebrandt F, Gefeller O, Rascher W (2008). Cyclosporin A is superior to cyclophosphamide in children with steroid-resistant nephrotic syndrome-a randomized controlled multicentre trial by the Arbeitsgemeinschaft fur Padiatrische Nephrologie. Pediatr Nephrol.

[21] Ponticelli C, Rizzoni G, Edefonti A, Altieri P, Rivolta E, Rinaldi S (1993). A randomized trial of cyclosporine in steroid-resistant idiopathic nephrotic syndrome. Kidney Int.

[22] Valverde S, Hernandez A, Velasquez L, Romero B, Mendoza A, Ramon G (2010). Efficacy of prednisone-tacrolimus vs. prednisone - Cyclosporine in steroid-resistant nephrotic syndrome. Pediatr Nephrol.

[23] Jiang X, Shen W, Xu X, Shen X, Li Y, He Q (2018). Immunosuppressive therapy for steroid-resistant nephrotic syndrome: a Bayesian network meta-analysis of randomized controlled studies. Clin Exp Nephrol.

[24] Hodson EM, Wong SC, Willis NS, Craig JC (2016). Interventions for idiopathic steroid-resistant nephrotic syndrome in children. Cochrane database of Syst Rev.

